# Lung Segmentation in 4D CT Volumes Based on Robust Active Shape Model Matching

**DOI:** 10.1155/2015/125648

**Published:** 2015-10-08

**Authors:** Gurman Gill, Reinhard R. Beichel

**Affiliations:** ^1^Department of Electrical and Computer Engineering, The University of Iowa, Iowa City, IA 52242, USA; ^2^The Iowa Institute for Biomedical Imaging, The University of Iowa, Iowa City, IA 52242, USA; ^3^Department of Internal Medicine, The University of Iowa, Iowa City, IA 52242, USA

## Abstract

Dynamic and longitudinal lung CT imaging produce 4D lung image data sets, enabling applications like radiation treatment planning or assessment of response to treatment of lung diseases. In this paper, we present a 4D lung segmentation method that mutually utilizes all individual CT volumes to derive segmentations for each CT data set. Our approach is based on a 3D robust active shape model and extends it to fully utilize 4D lung image data sets. This yields an initial segmentation for the 4D volume, which is then refined by using a 4D optimal surface finding algorithm. The approach was evaluated on a diverse set of 152 CT scans of normal and diseased lungs, consisting of total lung capacity and functional residual capacity scan pairs. In addition, a comparison to a 3D segmentation method and a registration based 4D lung segmentation approach was performed. The proposed 4D method obtained an average Dice coefficient of 0.9773 ± 0.0254, which was statistically significantly better (*p* value ≪0.001) than the 3D method (0.9659 ± 0.0517). Compared to the registration based 4D method, our method obtained better or similar performance, but was 58.6% faster. Also, the method can be easily expanded to process 4D CT data sets consisting of several volumes.

## 1. Introduction

Applications like lung cancer radiotherapy planning [1], assessment of lung diseases like COPD [2], or dynamic lung ventilation studies [3] require the acquisition and subsequent analysis of 4D lung CT scans (e.g., two lung scans at different respiratory states). Most quantitative analysis approaches utilize image registration methods [4, 5] for 4D analysis. In order to achieve accurate results and reduce computation time, registration is typically only performed within a lung mask. Thus, for such approaches, the segmentation of each lung CT volume acquired is a prerequisite. This can be accomplished by utilizing standard 3D lung segmentation methods like the ones proposed in [6–10], which assume a large density difference between air-filled lung parenchyma and surrounding objects/tissues. However, since 4D imaging is mainly performed for the assessment and/or treatment of lung diseases, such simple methods frequently fail to perform well. Recently, 3D segmentation methods have been developed to deal with this issue, including approaches that utilize an atlas-based segmentation-by-registration scheme [11], an error-correcting hybrid system [12], a shape “break-and-repair” strategy [13], and a 3D robust active shape model (RASM) [14, 15]. However, none of these approaches takes advantage of 4D lung CT scans and thus requires lungs to be segmented individually. This can be problematic, especially when segmenting pairs of total lung capacity (TLC) and functional residual capacity (FRC) lung scans, because (diseased) lungs at FRC are typically more difficult to segment than lungs imaged at TLC. Consequently, algorithms that simultaneously segment lungs in all available CT volumes are more promising.

Work on 4D lung segmentation techniques is scant. Wilms et al. [16] adopted a 4D statistical shape model that was originally developed by Perperidis et al. [17] for segmentation of gated cardiac image sequences. A limitation of this approach is that it is based on standard least squares active shape model (ASM) matching, which is known to be affected by outliers [14, 18]. Consequently, disease induced changes of lung tissue (e.g., density) or artifacts resulting from sorting algorithm errors in case of free-breathing CT lung imaging can adversely impact model matching.

**Figure 1:**
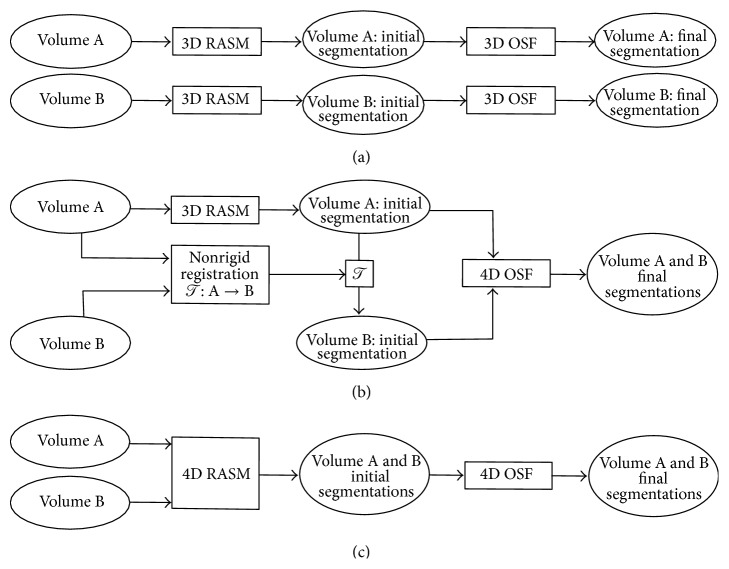
RASM-based approaches for segmenting lungs in a 4D CT volume (represented by 3D volumes A and B). (a) 3D segmentation method [14]. (b) Registration based 4D segmentation method [19]. (c) Proposed 4D segmentation approach with new 4D RASM (Section 3.1).

In our previous work, Sun et al. [19] introduced a 4D lung segmentation method based on 4D optimal surface finding (OSF). The approach requires a rough initial lung segmentation, which was obtained by applying a 3D RASM [14] to a TLC lung scan and transferring this segmentation by means of a nonrigid image registration to the corresponding FRC scan (Figure 1(b)). This approach has some potential shortcomings. First, the initial, rough segmentation step does not take full advantage of the available 4D CT data. Consequently, if the initial lung segmentation is inaccurate, the error is propagated to the other volume by the algorithm. Second, for many applications (e.g., segmentation of longitudinal TLC volumes), it is not obvious which volume should be utilized for 3D RASM segmentation to achieve good segmentation performance. Third, the registration step is quite time-consuming.

In this paper, we address these limitations by proposing a new 4D RASM model matching step that replaces the combination of single 3D RASM segmentation and subsequent registration to other volumes as proposed by Sun et al. [19]. In addition, we provide an extensive study, comparing the 3D base method published by Sun et al. [14] applied to each CT scan independently (Figure 1(a)), the registration based 4D version [19] (Figure 1(b)), and the proposed approach (Figure 1(c)) on a diverse set of 4D CT data, consisting of TLC and FRC scan pairs of normal and diseased lungs.

## 2. Prior Work

The proposed method extends our 3D RASM method [14] to mutual segmentation of lungs in 4D CT data. Thus, we briefly outline the RASM fitting process for a single 3D volume.

The RASM consists of a point distribution model (PDM) that captures the variation in lung shapes and a robust matching approach that iteratively fits the model to a lung CT scan to perform a segmentation. The PDM is constructed separately for left and right lungs from *N* lung volume training data sets that have *m* corresponding points (landmarks) [14]. An instance of a left or right lung shape is generated from the corresponding PDM by the linear model

(1) $$\begin{array}{c} x=\bar{x}+Pb, \end{array}$$

where $\bar{x}$ is the mean lung shape vector, *𝒫* denotes the shape eigenvector matrix, and **b** represents the shape coefficients.

The matching process begins with automatically placing the mean lung shape $\bar{x}$ in the target CT volume based on a ribs detection step [14]. The model shape points are then updated to **y** based on a gradient based cost function. For this purpose, a robust matching step is utilized to prevent that outliers are used during the model matching process. It is based on a robust PCA coefficient estimation method, which utilizes subsets of landmark points *ω*_*i*,*j*_ and a voting scheme [14]. During matching, for each of these subsets a reconstruction error *e*_*ω*_*i*,*j*__ is calculated, which is then being used to determine the inliers update points $\tilde{y}$ of **y** (note the notation for specifying inliers using $\tilde{\;}$; e.g., $\tilde{y}$ corresponds to the set **y**_{*p*_*i*_=1}_ in [14] and is used here instead for the sake of clarity). Subsequently, $\tilde{y}$ is utilized to update the shape coefficients by calculating

(2) $$\begin{array}{c} b={\tilde{P}}^{T}\left(\;T\tilde{y}-\tilde{\bar{x}}\;\right), \end{array}$$

where *𝒯* is the pose transformation matrix for mapping points from target image coordinate frame to model coordinate frame and $\tilde{\bar{x}}$ denotes the points corresponding to inliers in the mean lung shape $\bar{x}$. $\tilde{𝒫}$ refers to the columns corresponding to inliers in the shape eigenvector matrix *𝒫*. A new instance of the model is calculated using (1), which is transformed to the image space by *𝒯* ^−1^. The model shape points are then iteratively updated until convergence.

Once the robust matching process is finished, the resulting RASM segmentation is used as an initial shape for a graph-based optimal surface finding (OSF) algorithm to further refine the segmentation [14] (Figure 1(a)).

## 3. Methods

**Figure 2:**
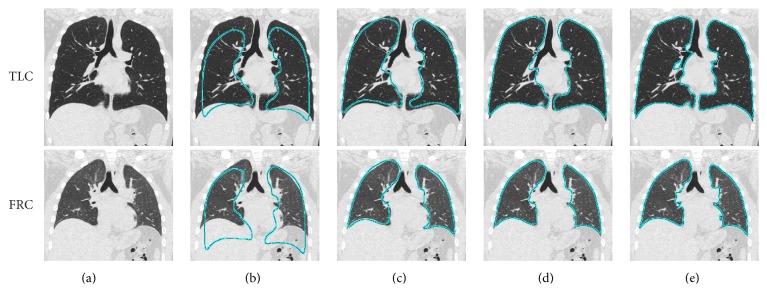
Illustration of intermediate and final results of the proposed 4D lung segmentation approach. (a) 4D CT data consisting of a TLC and FRC lung scan pair to be segmented. (b) Model initialization. (c-d) 4D RASM segmentation with (c) mutual segmentations and (d) result after the constrained adaptation step. (e) 4D OSF segmentation result.

Our method for generating a 4D lung segmentation (Figure 1(c)) is based on fitting a 3D RASM mutually to 4D volume data (Section 3.1), followed by a 4D OSF segmentation step (Section 3.2). The results of all the different processing stages are depicted in Figure 2. Below, we describe the segmentation process in detail for a TLC and FRC lung scan pair, but the approach would also work for other respiratory states or longitudinal scans and can be expanded to more than two lung volumes.

In addition to the main processing steps described in Sections 3.1 and 3.2, the following two preprocessing steps are performed. First, a modified system of the airway tree segmentation method [20] is utilized to extract the trachea and main bronchi, which are then dilated using a radius of 2 voxels. These locations are assigned a value of 50 HU in order to make them unattractive for RASM and OSF segmentation. Second, an overlap between left and right lung segmentations is avoided by detecting the thin tissue layer between the lungs, as described by Gill et al. [21].

### 3.1. 4D RASM Segmentation

**Figure 3:**
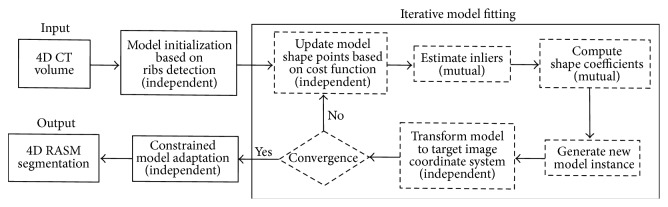
Flowchart showing the steps involved in fitting the model to 4D CT data. The independent and mutual steps are identified in the iterative model fitting process.

For model-based segmentation, a lung PDM is constructed from 75 TLC and 75 FRC normal lung CT scan pairs, which are not part of the image data utilized for method evaluation (Section 4.1). Note that model building is done separately for right and left lungs. Utilizing the right or left PDM, 4D RASM segmentation consists of the following main processing steps (Figure 3).

*(a) Model Initialization.* The mean lung model $\bar{x}$ is placed independently in the target TLC and FRC volumes (Figure 2(b)). For this purpose, a ribs detection method [14] is applied on the respective volumes.

*(b) Iterative Model Fitting.* The matching steps (i) to (v) given below are repeated for 90 iterations, which are sufficient to achieve model convergence (Figure 2(c)). Alternatively, a convergence criterion could be used.

(i) *Updating Shape Points.* Utilizing a gray-value gradient based cost function [14] of TLC and FRC volumes, the model shape points are independently updated in the TLC and FRC volumes, resulting in **y**_tlc_ and **y**_frc_, respectively.
(ii) *Robustly Estimating Mutual Inlier Update Points.* Update point sets **y**_tlc_ and **y**_frc_ are used to calculate *e*_*ω*_*i*,*j*__^tlc^ and *e*_*ω*_*i*,*j*__^frc^, respectively, which is similar to that described in [14]. However, after this step, a mutual reconstruction error is calculated with
  (3) $$\begin{array}{c} e_{\omega_{i,j}}^{\text{mutual}}=\frac{e_{\omega_{i,j}}^{\text{tlc}}+e_{\omega_{i,j}}^{\text{frc}}}{2} \end{array}$$
  to enable mutual inlier estimation. Thus, *e*_*ω*_*i*,*j*__^mutual^ is used in the voting scheme described in [14] instead of individual reconstruction errors *e*_*ω*_*i*,*j*__^tlc^ or *e*_*ω*_*i*,*j*__^frc^. The outcomes of the voting process are inlier update point sets ${\tilde{y}}_{tlc}$ and ${\tilde{y}}_{frc}$. Note that, while ${\tilde{y}}_{tlc}$ and ${\tilde{y}}_{frc}$ are different, they have the same cardinality and correspond to the same landmark points of the lung PDM.
(iii) *Computing Mutual Shape Coefficients.* The inlier point sets ${\tilde{y}}_{tlc}$ and ${\tilde{y}}_{frc}$ are independently transformed to the model coordinate frame by using pose transformation matrices *𝒯*_tlc_ and *𝒯*_frc_, respectively. Each transformation is derived from a Procrustes analysis between inlier sets (${\tilde{y}}_{tlc}$ and ${\tilde{y}}_{frc}$) and corresponding mean model ($\tilde{\bar{x}}$) in model coordinate space. The shape coefficients **b** ^mutual^ are computed using the average of the transformed inliers
  (4) $$\begin{array}{c} b^{\text{mutual}}={\tilde{P}}^{T}\left(\;\frac{T_{\text{tlc}}{\tilde{y}}_{\text{tlc}}+T_{\text{frc}}{\tilde{y}}_{\text{frc}}}{2}-\tilde{\bar{x}}\;\right). \end{array}$$
(iv) *Generating a New Model Instance.* A new instance of the model, which is used to represent the lung in TLC and FRC scans, is calculated using (1) and **b** ^mutual^.
(v) *Transforming the Model.* The model is transformed back to TLC and FRC volumes using *𝒯*_tlc_^−1^ and *𝒯*_frc_^−1^, respectively.

*(c) Constrained Model Adaptation.* After the 4D fitting process converges, a single lung shape with individual transformation matrices *𝒯*_tlc_ and *𝒯*_frc_ results, which matches the lungs in TLC and FRC scans. However, the transformations only account for isotropic scaling. Thus, the fitted models will not be perfectly aligned with the image data, because the difference in TLC and FRC lung shapes cannot be explained by an isotropic scale factor. To obtain a better alignment, we subsequently allow the shape coefficients to individually adapt to the target images by continuing the RASM fitting process independently in both volumes for ten iterations (Figure 2(d)). Note that this adaptation is done in a constrained manner, only allowing a subset of model coefficients to change within certain limits to avoid major divergence of TLC and FRC models. The subset of model coefficients (sorted in decreasing order of their eigenvalues) is defined by the coefficients whose eigenvalues account for 80% of shape variation. In our case, this resulted in a set of 22 coefficients out of 150, which were allowed to change by a maximum of 0.5 times the standard deviation *σ* in the respective eigenvalues. Thus, the final shape coefficients **b** ^adapted^ after the individual adaptation step for the TLC or FRC volume are limited to

(5) $$\begin{array}{c} b^{\text{mutual}}\left(\;l\;\right)\pm 0.5\sigma \left(\;l\;\right)\;\text{if }l\leq 22\\ b^{\text{mutual}}\left(\;l\;\right)\;otherwise. \end{array}$$

The parameters constraining the model were selected conservatively, and we found that small parameter variations have little impact on the overall lung segmentation.

### 3.2. 4D OSF Segmentation

After the initial model-based segmentations are created for TLC and FRC volumes, they are refined using the 4D OSF method [19], resulting in the final 4D lung segmentation (Figure 2(e)). For this purpose, the same parameter settings as proposed by Sun et al. [19] were utilized.

## 4. Evaluation

### 4.1. Image Data

For evaluation, 152 multidetector computed tomography (MDCT) thorax scans of lungs from 4 different sets *S*_normal_, *S*_asthma_, *S*_COPD_, and *S*_mix_ with no significant abnormalities (normal), asthma (both severe and nonsevere), chronic obstructive pulmonary disease (COPD, GOLD 1 to 4), and a mixture of different lung diseases, respectively, were utilized. The total number of scans in sets *S*_normal_, *S*_asthma_, *S*_COPD_, and *S*_mix_ were 40, 36, 36, and 40, respectively. All the four sets contained pairs of TLC (volume A) and FRC (volume B) images. The image sizes varied from 512 × 512 × 351 to 512 × 512 × 781 voxels with a mean size of 512 × 512 × 580 voxels. The slice thickness of images ranged from 0.5 to 0.63 mm (mean: 0.52 mm) and the in-plane resolution from 0.49 × 0.49 to 0.91 × 0.91 mm (mean: 0.64 × 0.64 mm).

### 4.2. Experimental Setup

**Figure 4:**
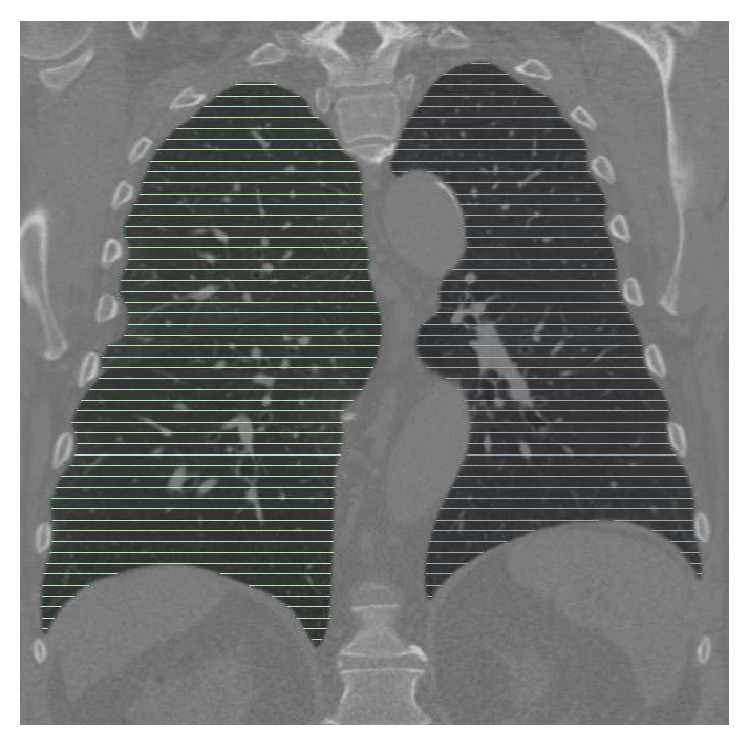
Example showing the location and density of axial reference segmentations in relation to lung anatomy.

For all test data sets, an independent reference standard was generated. Manual segmentation of a whole lung is time-consuming, and due to the large number of 152 test CT scans, we utilize a sampling approach, which is similar to that utilized in [6, 11, 12], to reduce the substantial effort required for manual inspection and segmentation. Thus, for every tenth axial slice, a trained expert generated a reference segmentation under the supervision of a pulmonologist, resulting in a dense sampling of the lung volume with between 41 and 64 labeled slices for each data set (Figure 4). The same sampling approach was applied to the segmentation result to be evaluated. Based on the sampled volumes, the Dice coefficient *D* [22] was calculated. In addition, the mean unsigned distance error *d* [22] was computed with respect to the reference in all axial slices where a reference standard and segmentation result were both available. Subsequently, the average of all these locations was calculated per data set and reported.

In the following sections, the proposed method (Figure 1(c)) will be denoted by *M*_4D_. In addition, two other methods will be utilized for comparison. *M*_3D_ will be utilized to denote the 3D approach proposed by Sun et al. [14] (Figure 1(a)). The 4D method of Sun et al. [19] (Figure 1(b)) was used in two variants; the variant where volume A (TLC) is registered to volume B (FRC) will be denoted by *M*_4DregAB_, and the variant where volume B (FRC) is registered to volume A (TLC) will be denoted by *M*_4DregBA_. Investigating these two variants allows us to assess and compare performance in situations with different but unknown respiratory state (e.g., longitudinal lung image data). For all methods utilized, the standard parameter setting as described in respective papers was used. Unless otherwise mentioned, all reported results refer to the final (OSF) segmentation.

A paired permutation test [23] was utilized for determining statistical significance, because it does not make assumptions about the distribution of the underlying data and a paired *t*-test or paired signed rank test was not applicable to our data.

## 5. Results

### 5.1. Segmentation Performance

Our novel 4D RASM matching approach (without final OSF segmentation step) showed an average Dice coefficient of 0.9468 ± 0.0318. In contrast, the standard 3D RASM approach resulted in an average Dice coefficient of 0.9391 ± 0.0525. The 4D RASM showed a statistically significant improvement (*p* value ≪0.001) compared against 3D RASM.

**Figure 5:**
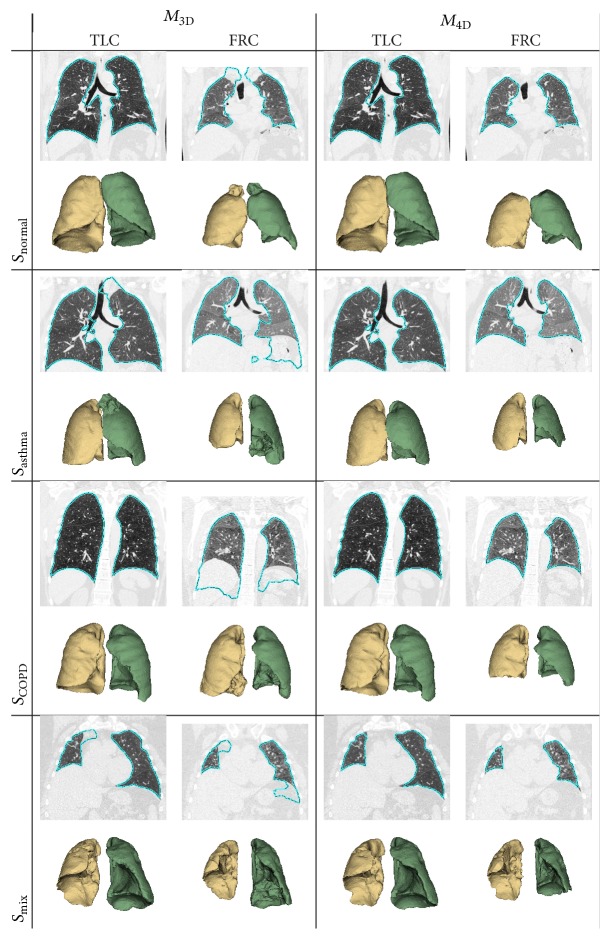
Comparison of segmentation results generated with methods *M*_3D_ and *M*_4D_. Coronal CT cross sections with marked lung boundaries in combination with corresponding 3D lung mesh models are shown to enable locating and assessing segmentation errors as well as differences between results.

Tables 1 and 2 summarize the resulting final (OSF) segmentation performance with corresponding *p* values, comparing results of *M*_3D_ and *M*_4D_. In both tables, *M*_4D_ shows statistically significant improvement in each data set and for TLC and FRC scans.  Figure 5 depicts some examples of segmentations (one from each test data set) obtained by methods *M*_3D_ and *M*_4D_.

**Figure 6:**
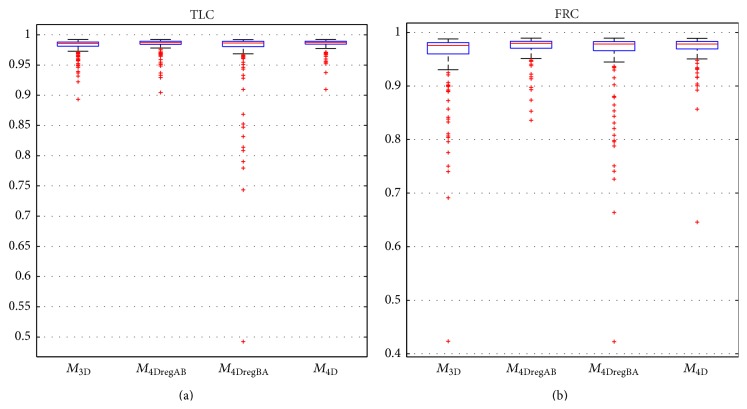
Box plots of Dice coefficients for methods *M*_3D_, *M*_4DregAB_, *M*_4DregBA_, and *M*_4D_ on (a) TLC and (b) FRC lung scans.

**Figure 7:**
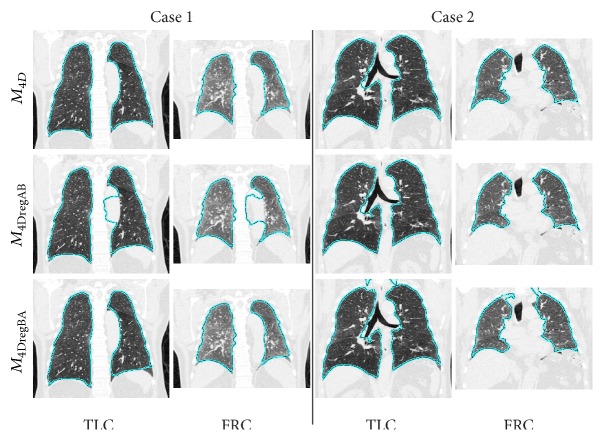
Examples of segmentation results generated with methods *M*_4D_, *M*_4DregAB_, and *M*_4DregBA_.

Tables 3 and 4 compare the final lung segmentation Dice coefficient of *M*_4D_ to *M*_4DregAB_ and *M*_4DregBA_, respectively. Overall, *M*_4D_ and *M*_4DregAB_ were found to be equivalent (no statistically significant difference), but *M*_4D_ was found to be significantly better compared to *M*_4DregBA_.  Figure 6 provides a comparison of final Dice coefficients in form of box plots for all methods and separated by respiratory state. A comparison of results generated with *M*_4D_, *M*_4DregAB_, and *M*_4DregBA_ is shown in Figure 7.

### 5.2. Computing Time

Segmentation with *M*_3D_ took 13.21 minutes on average for TLC and FRC data sets combined (TLC: 6.73 minutes, FRC: 6.48 minutes). Method *M*_4D_ required 12.22 minutes per 4D case, on average. Compared to *M*_3D_, the reduction in computing time was primarily achieved due to synergies of 4D processing. Approaches *M*_4DregAB_ and *M*_4DregAB_ took 29.49 minutes per 4D case, on average, where the registration procedure contributed to about 20 minutes of computing time.

## 6. Discussion

The main advantage of our 4D approach *M*_4D_ is that it utilizes both lung volumes acquired at different respirator states for segmentation during all main processing stages, which is in contrast to the standard *M*_3D_ method and 4D variants *M*_4DregAB_ and *M*_4DregBA_. The results presented in Section 5 clearly demonstrate this advantage.

### 6.1. Comparison of *M*_4D_ with *M*_3D_

When compared to the 3D variant, statistically significant lung segmentation performance improvements, independent of test set, respiratory state, and performance metric, were observed (Tables 1 and 2). This is also clearly demonstrated by the examples shown in Figure 5. As shown in Tables 1 and 2, the observed gain in segmentation accuracy with *M*_4D_ was larger for cases with lung disease (test sets *S*_asthma_, *S*_COPD_, and *S*_mix_) compared to normal cases (test set *S*_normal_). The better segmentation performance of *M*_4D_ is expected, because it addresses several weaknesses of *M*_3D_ like problems with model initialization, which can cause the model to converge locally to other structures than lung boundaries. As Figure 6 as well as Tables 1 and 2 show, gains achieved with *M*_4D_ are higher for lungs imaged at FRC, which are generally more difficult to segment. Also, 4D processing reduces the computing time by 7.5% compared to sequential 3D processing.

### 6.2. Comparison of *M*_4D_ with *M*_4DregAB_ and *M*_4DregBA_

Overall, segmentation performance of *M*_4D_ and *M*_4DregAB_ was found to be equivalent (Table 3), while the comparison between *M*_4D_ and *M*_4DregBA_ (Table 4) showed a statistically significant improvement for our proposed *M*_4D_ approach. Also, compared to *M*_4DregAB_ and *M*_4DregBA_, our *M*_4D_ method showed a reduction in computing time by 58.6%. Thus, it is preferable to *M*_4DregAB_ and *M*_4DregBA_, especially when the exact respiratory states are unknown and picking a lung scan with lower lung volume (exhale) as starting point (Volume A in Figure 1(b)) could potentially adversely impact segmentation performance. Figure 7 depicts examples where either *M*_4DregAB_ or *M*_4DregBA_ produces a local segmentation error, but *M*_4D_ avoids such problems.

### 6.3. Possible Improvements and Extensions

As can be seen in Figure 6, the proposed approach reduces the number and/or severity of outlier cases. However, some room for improvement still exists, which we will address in future work. For example, a better FRC model initialization would help in further improving overall segmentation performance of 3D and 4D methods, but we expect that *M*_4D_ would still perform better, because it utilizes both scans for model matching, which offers increased robustness.

**Figure 8:**
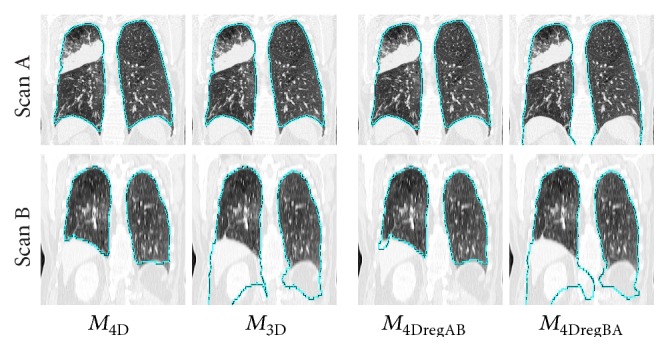
Comparison of results of methods *M*_4D_, *M*_3D_, *M*_4DregAB_, and *M*_4DregBA_ on a longitudinal CT volume pair. The time between scans A and B was 19 months, and the imaging protocol was quite different.

Our method can be expanded to handle processing of more than two lungs scans at the same time. This can be done by extending (3) and (4) accordingly. Another advantage is that *M*_4D_ does not require any prior knowledge of the breathing state of the lungs in individual CT scans, because it does not make any assumptions about respiratory state (e.g., breathing sequence). An example for processing longitudinal lung CT scans in the context of cancer treatment planning/assessment is provided in Figure 8. Note that, due to lung disease (cancer), patient compliance with the utilized imaging protocol (e.g., acquisition at TLC) cannot be assumed.

## 7. Conclusions

In this paper, we have presented a 4D lung segmentation approach that utilizes a new 4D robust active shape model matching method and provided an evaluation of this method on a diverse set of 76 TLC and FRC lung scan pairs. In addition, a detailed comparison with its 3D lung segmentation counterpart as well as two variants of 4D registration based lung segmentation methods was performed, demonstrating the advantages of our approach in terms of segmentation performance and/or computing time. By avoiding any assumptions about the respiratory state of the imaged lungs, our approach provides flexibility and is applicable to pairs of TLC and FRC scans, other dynamic 4D lung CT scans, and longitudinal CT studies. Thus, the developed method is suited for applications like cancer treatment planning or assessment of other lung diseases like emphysema.

## Figures and Tables

**Table 1:** Dice coefficient *D* for methods *M*_3D_ and *M*_4D_. Statistically significant *p* values are marked with *∗*.

Set	*M*_3D_	*M*_4D_	*p* value
*S*_normal_	0.9822 ± 0.0129	**0.9850 ± 0.0058**	≪0.001^*∗*^
*S*_asthma_	0.9667 ± 0.0437	**0.9789 ± 0.0108**	≪0.001^*∗*^
*S*_COPD_	0.9685 ± 0.0514	**0.9839 ± 0.0068**	≪0.001^*∗*^
*S*_mix_	0.9467 ± 0.0732	**0.9623 ± 0.0444**	0.012^*∗*^

TLC	0.9808 ± 0.0142	**0.9846 ± 0.0103**	≪0.001^*∗*^
FRC	0.9510 ± 0.0687	**0.9700 ± 0.0329**	≪0.001^*∗*^

ALL	0.9659 ± 0.0517	**0.9773 ± 0.0254**	≪0.001^*∗*^

**Table 2:** Mean unsigned distance error *d* in millimeters for methods *M*_3D_ and *M*_4D_. Statistically significant *p* values are marked with *∗*.

Set	*M*_3D_	*M*_4D_	*p* value
*S*_normal_	1.20 ± 0.77	**0.89 ± 0.44**	≪0.001^*∗*^
*S*_asthma_	1.68 ± 1.25	**1.14 ± 0.50**	≪0.001^*∗*^
*S*_COPD_	1.66 ± 1.90	**1.00 ± 0.56**	≪0.001^*∗*^
*S*_mix_	2.77 ± 4.42	**1.81 ± 1.45**	≪0.001^*∗*^

TLC	1.16 ± 0.68	**0.88 ± 0.53**	≪0.001^*∗*^
FRC	2.52 ± 3.50	**1.56 ± 1.11**	≪0.001^*∗*^

ALL	1.84 ± 2.61	**1.22 ± 0.93**	≪0.001^*∗*^

**Table 3:** Dice coefficient *D* for methods *M*_4DregAB_ and *M*_4D_. Statistically significant *p* values are marked with *∗*.

Set	*M*_4DRegAB_	*M*_4D_	*p* value
TLC	0.9834 ± 0.0127	**0.9846 ± 0.0103**	8.92*e* − 03^*∗*^
FRC	**0.9718 ± 0.0233**	0.9700 ± 0.0329	2.07*e* − 01

ALL	**0.9776 ± 0.0196**	0.9773 ± 0.0254	7.97*e* − 01

**Table 4:** Dice coefficient *D* for methods *M*_4DregBA_ and *M*_4D_. Statistically significant *p* values are marked with *∗*.

Set	*M*_4DregBA_	*M*_4D_	*p* value
TLC	0.9687 ± 0.0579	**0.9846 ± 0.0103**	≪0.001^*∗*^
FRC	0.9539 ± 0.0711	**0.9700 ± 0.0329**	≪0.001^*∗*^

ALL	0.9613 ± 0.0651	**0.9773 ± 0.0254**	≪0.001^*∗*^
